# Identification of RNA binding motifs in the R2 retrotransposon-encoded reverse transcriptase

**DOI:** 10.1093/nar/gku514

**Published:** 2014-06-21

**Authors:** Varuni K. Jamburuthugoda, Thomas H. Eickbush

**Affiliations:** Department of Biology, University of Rochester, Rochester, NY 14627, USA

## Abstract

R2 non-LTR retrotransposons insert at a specific site in the 28S rRNA genes of many animal phyla. R2 elements encode a single polypeptide with reverse transcriptase, endonuclease and nucleic acid binding domains. Integration involves separate cleavage of the two DNA strands at the target site and utilization of the released 3′ ends to prime DNA synthesis. Critical to this integration is the ability of the protein to specifically bind 3′ and 5′ regions of the R2 RNA. In this report, alanine mutations in two conserved motifs N-terminal to the reverse transcriptase domain were generated and shown to result in proteins that retained the ability to cleave the first strand of the DNA target, to reverse transcribe RNA from an annealed primer and to displace annealed RNA when using DNA as a template. However, the mutant proteins had greatly reduced ability to bind 3′ and 5′ RNA in mobility shift assays, use the DNA target to prime reverse transcription and conduct second-strand DNA cleavage. These motifs thus appear to participate in all activities of the R2 protein known to require specific RNA binding. The similarity of these R2 RNA binding motifs to those of telomerase and group II introns is discussed.

## INTRODUCTION

**Figure 1.**
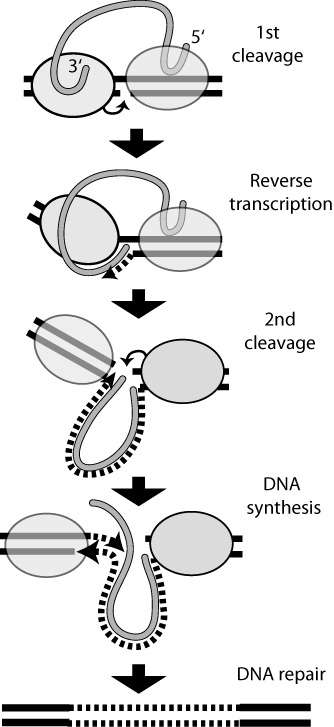
TPRT model for the integration of R2 elements (3,4). DNA target site, solid black line; R2 RNA transcript, solid gray line; newly synthesized R2 DNA, dashed black line; gray oval, R2 protein. Association of the R2 protein with the 3′ end of the R2 transcript directs DNA binding of the protein upstream of the 28S rRNA gene insertion site. This protein cleaves the bottom strand of DNA and uses the released 3′ end to prime reverse transcription of the R2 transcript (cDNA synthesis) directly onto the target site. Association of the R2 protein with the 5′ end of the R2 transcript directs DNA binding of the protein downstream of the insertion site. This protein cleaves the top strand of DNA and uses the released 3′ end to prime synthesis of the second DNA strand displacing the RNA strand while using the cDNA as template. Finally, DNA repair seals the nicks remaining at each end of the insertion.

Non-LTR retrotransposable elements, also known as long interspersed nuclear elements (LINEs), are highly abundant genomic parasites of eukaryotes. One of the best-characterized members of this class is R2, an element which exclusively inserts into a highly conserved sequence of the 28S rRNA genes (1,2). The extreme sequence specificity of R2 for a site in the 28S rRNA genes has enabled detailed studies of its retrotransposition mechanism (3,4). These studies have suggested that integration involves symmetrical reactions by R2 protein subunits bound upstream and downstream of the insertion site (Figure 1). Protein bound the 3′ end of the R2 RNA transcript cleaves one strand of the DNA target and uses the cleaved end to prime reverse transcription of the R2 transcript. Protein bound to the 5′ end of the RNA transcript cleaves the second DNA strand and again uses the released DNA end to prime synthesis of the second DNA strand. This integration mechanism has been termed target primed reverse transcription (TPRT) (3).

The reverse transcriptase encoded by R2 (R2-RT) has unusual properties when compared to the RTs of retroviruses. First, priming of DNA synthesis from the 3′ hydroxyl exposed by the DNA cleavage does not require complementarities between the DNA target and the RNA template (5,6). Second, R2-RT is highly processive, presumably because release of the RT from the RNA template would result in a truncated insertion (7). Third, upon reaching the end of an RNA template the R2-RT can add up to 5 non-templated nucleotides and use microhomology to jump to another RNA or DNA template (8). Finally, R2-RT can use the DNA of an RNA:DNA heteroduplex as template, displacing the RNA strand (9,10). One property that R2-RT shares with retroviral RT is the ability to extend past nucleotide mismatches resulting in an error rate similar to that of the RT from HIV-1 (11).

In addition to the specificity of the R2 endonuclease for the 28S gene target site, R2-RT has high specificity when binding RNA. The only RNA sequences utilized in the TPRT reaction are those that contain the 3′ untranslated region (3′ UTR) of R2 (5). This 3′ UTR RNA can be folded into a precise structure that has been shown to be conserved across related species (12,13). While enzymatic and DNA binding domains of the R2 protein have been previously characterized, nothing is known of the location or number of the RNA binding domains. Two model systems with potential clues to identifying the RNA binding domains in R2 are mobile group II introns and telomerase. Mobile group II introns are self-splicing retroelements found in bacteria and organellar genomes (14). These introns encode an enzymatically active RNA (ribozyme), which catalyses its own splicing from a co-transcript, and reverse splicing of that intron into a new DNA target site. These introns also encode a protein, which helps to fold the intron RNA (maturase), cleave the target site (endonuclease) and reverse transcribe the reverse-spliced RNA template using the new target site as primer (TPRT) (15). While telomerase is not encoded by a mobile element, this protein (TERT) binds a cellular RNA (TER) and catalyzes a reaction similar to a TPRT reaction (16). Namely, the 3′ end of a chromosome is used to repeatedly prime reverse transcription of a short sequence of TER, thereby, forming the tandemly repeated telomeric sequences found in most eukaryotes.

Therefore, the R2 protein, group II intron protein and telomerase share the property of specifically binding an RNA molecule that will be used as a template for reverse transcription primed by the 3′ end of a DNA strand. Critical RNA binding domains for both group II introns and telomerase have been shown to be immediately N-terminal to the fingers region of their reverse transcriptase domain (17–19). In this report, we show that the R2 protein also encodes RNA binding motifs immediately N-terminal of its RT domain. Mutations of these motifs do not affect recognition and first strand cleavage of the DNA target site or the basic properties of the RT, but they do affect the ability of the enzyme to conduct the TPRT reaction, bind the 3′ or 5′ end of R2 RNA and cleave the second DNA strand.

## MATERIALS AND METHODS

### Mutagenesis and purification of wild type and mutant R2 proteins

Alanine replacement mutations of the *Bombyx mori* R2 expression construct pR260 (3) in the -1 region (R310A, R311A, Q318A) and in the 0 region (G408A, D410A) were generated by the QuickChange Lightning Multi Site-Directed Mutagenesis kit (Stratagene) using mutagenic primers with point mutations in the appropriate codons. Mutant constructs were sequenced, transformed into *Escherichia coli* JM109 and proteins purified as described previously (3,10) with the following modifications. Protein extracts from the high speed centrifugation were diluted to 0.3 M NaCl and loaded onto a 20 ml Q-Sepharose column, washed extensively and eluted with 0.5 M NaCl. Protein fractions of 1 ml volume were collected and tested for reverse transcriptase activity on poly (rA)/poly (dT)_13–18_ templates with α-^32^P-labeled dTTP (10). Aliquots from each fraction were also run on a 1% agarose gel stained with ethidium bromide to determine the fractions containing *E. coli* RNA. Fractions with high reverse transcriptase activity without co-purifying RNA were pooled and dialyzed against R2 storage buffer for 3 h at 4ºC. The protein was stored in 0.1% Triton X-100 and 0.1 mg/ml bovine serum albumen at −20°C. Protein concentrations were determined by silver staining (BioRad) of sodium dodecyl sulphate (SDS) polyacrylamide gels using bovine serum albumen as the protein concentration standard.

### Preparation of DNA and RNA substrates

A 110-bp fragment of the 28S rRNA gene was used as the target DNA substrate for all cleavage, TPRT and electrophoretic mobility shift assays (EMSA). This DNA substrate was generated by PCR amplification from clone pB109 (3) with a forward primer (5′ AATTCAAGCAAGCGCGG 3′) complementary to a region 50 bp upstream of R2 insertion site and reverse primer (5′ CTAAGGATCCCGTTAAT CCATTCATG 3′) complementary to a region 60 bp downstream of the R2 insertion site. The target DNA substrates were 5′ end-labeled on either the bottom strand or on both strands by end-labeling the reverse or both PCR primers using γ ^32^P-ATP (Perkin-Elmer/Life Sciences, 6000 mCi/mmol). PCR amplifications, gel purification and elution of target DNA was performed as described previously (20) with the final DNA pellet dissolved in water and stored at −20°C. The 320-single stranded DNA template was derived from the M13mp18 vector (Invitrogen) as described previously (10). R2 3′RNA and 5′RNA were generated *in vitro* using T7 RNA polymerase and purified as described previously (9,10). The 100 bp non-specific (NS) RNA contained only M13mp18 sequences also as described previously (10).

### DNA cleavage and TPRT assays

All assays were performed in a 13 μl volume containing 10 mM Tris-HCl (pH 8.0), 200 mM NaCl, 5 mM MgCl_2_, 1 mM dithiothreitol, 0.1 mg/ml bovine serum albumin, 0.01% Triton X-100, 10%–12% glycerol with 4 ng labeled target DNA and variable amounts of R2 protein as described in the figure legends (4,20). The cleavage assays were performed by pre-incubating the R2 protein with 1 μg of RNase A or with 100 ng of 3′RNA, 5′RNA or non-specific RNA for 5 min at room temperature followed by incubating with target DNA for 20 min at 37°C. TPRT assays were performed by pre-incubating the R2 proteins at room temperature for 5 min with increasing amounts of 3′ RNA (12.5–100 ng), followed by incubating with target DNA and 25 μM of each dNTPs for 20 min at 37°C. Reactions were stopped by the addition of three volumes of 95% (v/v) ethanol containing 0.3 M sodium acetate pH 5.3 and 0.1% (w/v) SDS. After precipitation the products were resuspended and incubated at 95ºC for 5 min in 10 μl 40 mM ethylenediaminetetraacetic acid (EDTA), 99% formamide loading dye. The products were separated on 8% polyacrylamide-urea gels and quantified using a PhosphoImager (BioRad).

### Primer extension assays

The ability of wild type and mutant R2 proteins to extend DNA primers annealed to either RNA or DNA templates was assayed as described previously (11). Polymerization was initiated with 250 μM of each dNTPs with the amounts of substrate and protein used as described in the figure legends. Reactions were terminated and separated on 14% polyacrylamide-urea gels (11).

### Strand displacement assays

The primer, 5′ CTGGCGAAAGGGGGATGTGC 3′, was 5′ end labeled and annealed to a 320 nucleotide single stranded DNA template or this DNA template annealed to a 100 nt RNA block as described previously (10). Substrates were pre-incubated with the R2 proteins or T4 DNA polymerase (Fermentas) for 10 min at room temperature. Reactions were initiated by addition of 250 μM each dNTPs, incubated at 37ºC for 20 min and the reactions stopped by the addition of three volumes of 95% (v/v) ethanol containing 0.3 M sodium acetate pH 5.3 and 0.1% (w/v) SDS. Products were separated on 8% polyacrylamide-urea gels.

### Electrophoretic mobility shift assays

Reaction conditions were as described for the DNA cleavage and TPRT assays. Reactions were first pre-incubated with 1 μg of RNase A or 100 ng of 3′ or 5′ RNA for 5 min at room temperature followed by incubating with target DNA for 20 min at room temperature. Reactions were chilled on ice and run on 5% native polyacrylamide gel as described previously (4,20). EMSA assays were performed in either the presence or absence of 0.05 mM EDTA to inhibit/allow DNA cleavage. The non-specific competitor poly (dIdC) was added to 25 μg/ml in all reactions.

## RESULTS

### Conserved motifs upstream of the RT domain

**Figure 2.**
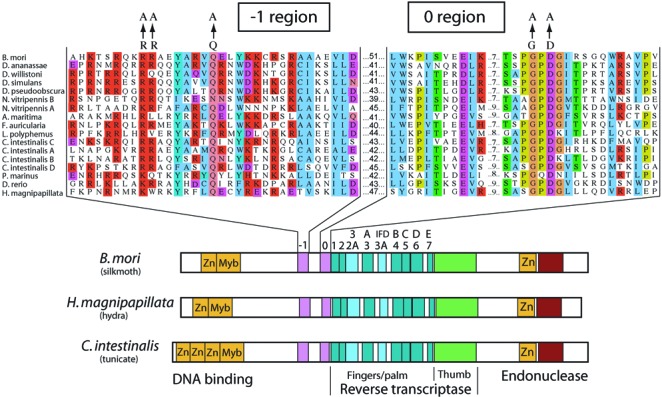
Location and sequence of the -1 and 0 regions in R2 elements. The R2 sequences shown at the top is an amino acid sequence alignment of the open reading frames immediately N-terminal to the common conserved motifs found in all reverse transcriptases. Positions with the same or chemically similar amino acids are shaded. The 17 R2 sequences come from diverse arthropods (10 sequences), the tunicate *Ciona intestinalis* (4 sequences), two vertebrates (*Petromyzon marinus* and *Danio rerio*) and a hydra (*Hydra magnipapillata*). The alanine replacements generated for the -1 and 0 mutant proteins are shown. At bottom are diagrams of the R2 proteins from three diverse metazoans indicating the similar structure of R2 proteins from all animals. The encoded protein contains N-terminal zinc finger (Zn) and c-myb domains (23), the various subdomains of all reverse transcriptases (21,25,32) and C-terminal zinc finger and endonuclease domains (22). An alternate labeling of reverse transcriptase domains frequently used for telomerases are shown above the more generic terms (16,18).

R2 elements are prevalent in most arthropod lineages (1) as well as other metazoans as diverse as vertebrates and hydra (2). All R2 elements contain a single open reading frame that encodes a 1100–1200 amino acid protein with similar conserved domains (Figure 2). The central one-third of the R2 protein contains the motifs that have been identified in the fingers, palm and thumb subdomains of various reverse transcriptases (21). C-terminal to the RT domain is a region encoding a zinc-finger motif and the active site of a restriction enzyme-like endonuclease (22). N-terminal to the RT domain is a DNA binding domain containing a c-myb and one to three zinc finger motifs (23). The region between the DNA binding domain and the RT domain is somewhat variable in length with only low levels of amino acid sequence conservation (24).

Because in both mobile group II introns and telomerase an RNA binding domain has been identified N-terminal to the fingers and palm subdomains of the RT (18,19), this region of the R2 Open Reading Frame (ORF) was compared in all available R2 sequences. The alignment in Figure 2 contains representative R2 sequences from 10 arthropods, two vertebrates and a hydra as well as multiple R2 lineages from a tunicate. Sequence conservation was detected in two regions, each ∼35 aa in length, separated by a 39–51 aa region with lower sequence identity. The region nearest the RT domain contained the highly conserved PGPDG sequence, which had previously been suggested to be part of the RT domain of all non-LTR retrotransposons (24,25). In these earlier reports, this motif was referred to as the 0 motif as it was N-terminal to the core motifs (labeled 1–7) that are conserved in all reverse transcriptases. The second region of the R2 proteins had less sequence constraints but contained a conserved interspersion of charged and hydrophobic residues and will be referred to as the -1 region.

Three conserved residues within region -1 and two residues in region 0 were individually mutated to alanine residues, and the mutant proteins tested for their ability to conduct the TPRT reaction with the 3′ UTR RNA of R2 (described below). While a slight reduction in product levels was sometimes detected (data not shown) all mutant proteins retained significant ability to conduct the TPRT reaction. More dramatic reductions were obtained by combining the three alanine mutations in the -1 region into one construct and the two mutations in the 0 region into a second construct. The activities of these two mutant proteins are described below and the proteins are referred to as the -1 and 0 region mutations.

### First strand DNA cleavage

**Figure 3.**
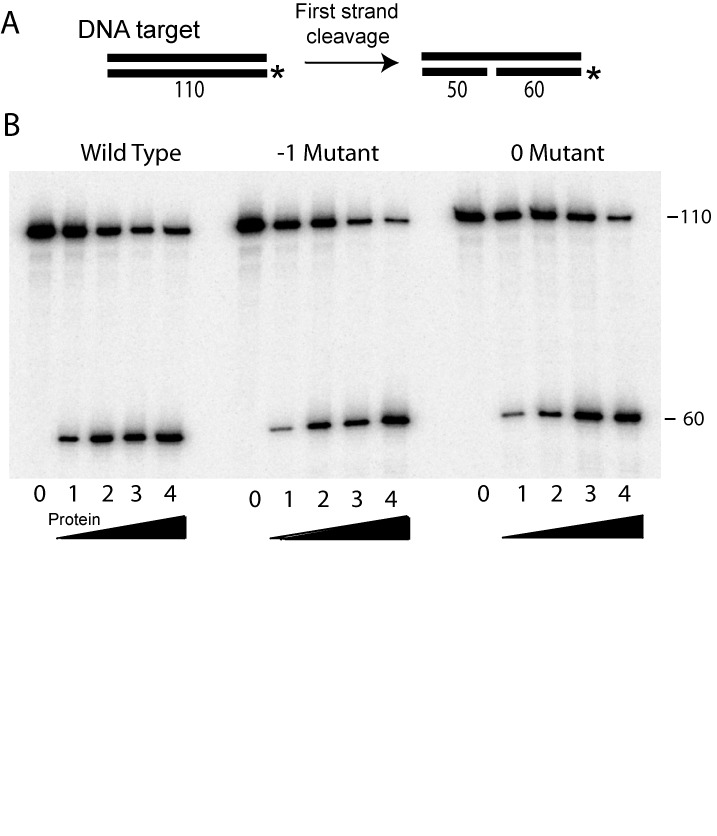
First strand DNA cleavage assay. (A) Diagram of the assay. The R2 protein is incubated with a 110 bp DNA fragment containing the 28S gene target site. The bottom stand (also called first strand) is 5′ end-labeled with P^32^. Cleavage generates a 60 nt labeled and a 50 nt unlabelled fragment. (B) Phosphoimager scan of cleavage reactions by the wild type, -1 and 0 mutant proteins separated under denaturing conditions on an 8% polyacrylamide-urea gel. For each reaction, 4 ng of target DNA was incubated with increasing amounts of protein for 20 min at 37°C. Lane 0, no protein; lane 1, 0.5 ng; lane 2, 1 ng; lane 3, 2 ng; lane 4, 4 ng.

To insure the mutations did not influence the overall structure or stability of the R2 protein, the -1 and 0 region mutant proteins were first tested for their ability to specifically cleave the first strand of the DNA target. Previous studies have shown that the R2 protein in the absence of RNA can cleave only one strand of the DNA target site (3). This first, or bottom, strand cleavage is used to prime the reverse transcription step. As shown in Figure 3A, the cleavage assay contained a 110-bp fragment of the 28S rRNA gene with the 5′ end of the bottom strand labeled (the DNA target site is drawn with the 5′ end of the 28S gene on the left). Because of the high affinity of the R2 protein for the DNA target site after cleavage, the R2 protein is capable of only a single cleavage cycle (26). The target DNA was incubated with increasing amounts of R2 protein (Figure 3B) for 30 min at 37ºC, and the cleavage products were separated on an 8% denaturing polyacrylamide gel. The wild-type and mutant proteins generated similar levels of the 60 nucleotide cleavage product, with ∼50% of target DNA cleaved with 2 ng of protein (lane 3). These data suggested that the 0 and -1 region mutations had minimal effect on the expression and purification of the R2 protein, its stability after purification and the ability of the protein to recognize and cleave the target DNA.

### Primer extension on RNA and DNA templates

**Figure 4.**
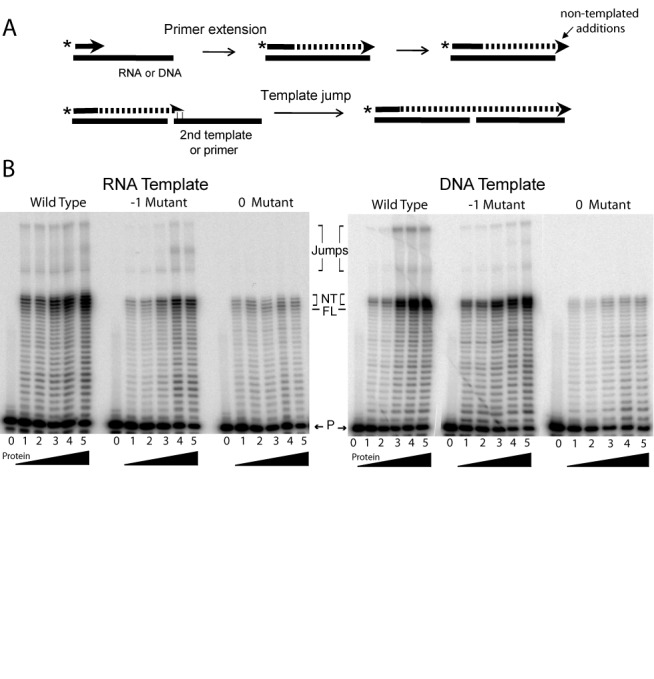
Primer extension assays on DNA and RNA substrates. (A) Diagram of the multiple products generated by the R2 polymerase in a primer extension assay. The reaction is started with a DNA primer annealed to the 3′ end of a DNA or RNA template. After extending to the end of the substrate, the R2 polymerase can add up to 5 non-templated nucleotides and then use microhomologies between this extension and the end of another substrate to jump to another template. (B) Phosphoimager scans of the products generated by the wild type, -1 and 0 mutant proteins. A 5′ end labeled 17-mer primer (P) was annealed to either a 38-mer RNA or DNA template. The template/primer (10 nM) was incubated with increasing amounts of protein for 15 min at 37ºC in the presence of 250 μM dNTPs (lane 0, no protein; lane 1, 0.25 ng; lane 2, 0.5 ng; lane 3, 1 ng; lane 4, 2 ng; lane 5, 4 ng). The extension products were analyzed on denaturing 14% polyacrylamide-urea gels. All three proteins added additional non-templated nucleotides (NT) beyond the band labeled full-length (FL) and the wild-type and -1 mutant proteins conducted template jumps to another template or the excess primer in the assay (bands labeled Jumps). The ladder of bands seen below the FL band corresponds to low levels of R2 protein dissociating from the template after each polymerization step (11).

To determine if the mutations affected the basic nucleotide polymerase activity of the protein, the 0 and -1 mutants were tested for their ability to extend a DNA primer annealed to a template. As diagramed in Figure 4A, the extension assays employed a 5′ end-labeled DNA primer annealed to either an RNA or DNA template. These primer extension assays also monitored two other properties of the wild-type R2-RT. First, upon reaching the end of the template, R2-RT adds 3 to 5 additional (non-templated) nucleotides (8). Second, the polymerase can use microhomology between the non-templated nucleotides and the end of another template to continue DNA synthesis. This activity, referred to as template jumping, can occur with both the excess primer and the single-stranded RNA or DNA template present in the assay (8).

As shown in Figure 4B, the -1 and 0 mutant proteins were able to extend the primer and generate full-length (FL) products on both the RNA and DNA templates. By using the amount of bottom strand DNA cleavage to equilibrate the level of active R2 protein, the 0 region mutant protein generated on average ∼20% fewer FL extension products compared to wild type and the -1 mutant in three different protein preparations. Upon reaching the end of the template, both mutant proteins were similar to wild-type protein in their ability to add up to 5 non-templated nucleotides (bands labeled NT directly above the FL band). However, while the -1 mutant protein retained the ability to jump from the initial template to another template and continue polymerization, template jumps with the 0 mutant were reduced to less than 10% of the wild-type protein. These experiments suggest the mutations in the -1 region of the R2 protein had minimal affect on the functioning of the RT domain. On the other hand, mutations in the 0 region somewhat reduced the efficiency of simple extension reactions and more extensively reduced the ability of the RT to jump between templates.

### Strand displacement during synthesis

**Figure 5.**
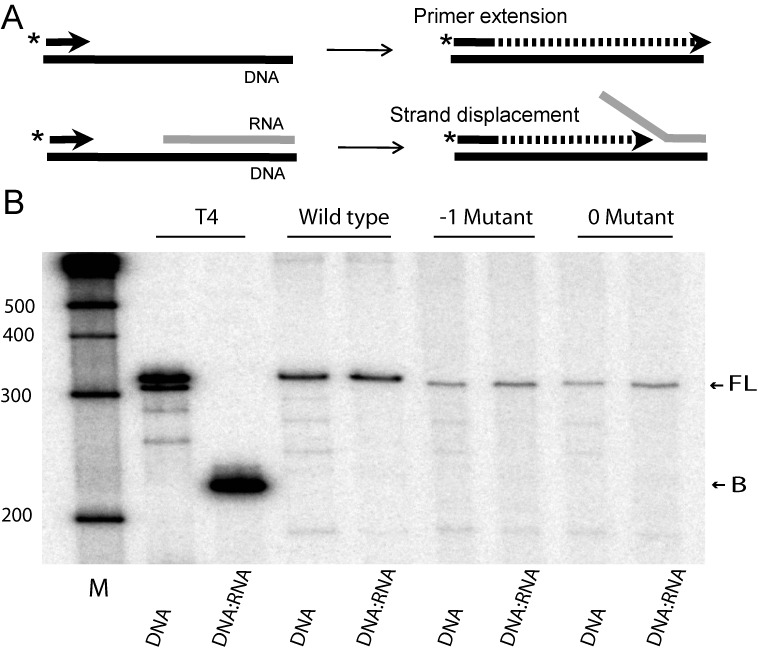
Assay to demonstrate that during DNA synthesis the R2 protein can displace an RNA strand annealed to a DNA template. (A) Diagram of the strand displacement assay. A primer was used to initiate synthesis either on a 320 nt DNA template or the same template with a 100 nt complementary RNA strand annealed to the opposite end. (B) Phosphoimager scan of the products of the strand displacement reactions for wild type, -1 and 0 mutant proteins separated on a denaturing 8% polyacrylamide-urea gel. T4 DNA polymerase, which has no ability to displace an annealed RNA strand, was used as a control to demonstrate that the DNA template was fully blocked by the annealed RNA.

Another unusual property of the R2 polymerase is its ability while synthesizing DNA to displace an RNA or DNA strand annealed to an RNA or DNA template (10). This activity is important in the R2 integration reaction because the R2 protein does not contain an RNase H domain (25), and no RNase H activity could be detected in *in vitro* assays (27). Therefore, in the TPRT reaction after reverse transcription by R2-RT (first stand synthesis), the RNA template remains annealed to the cDNA and must be displaced during second strand synthesis. To exam the ability of the -1 and 0 region mutants to conduct strand displacement, a 100-nt RNA strand (RNA block) was annealed to the 5′ end of a 320 nt DNA template. Polymerization was initiated with a 5′ end labeled primer annealed to the 3′ end of the DNA template (see Figure 5A). T4 polymerase, which lacks strand displacement activity (10), was used as a control to confirm that the DNA template was completely blocked by RNA annealed to the template DNA strand.

As shown in Figure 5B, in the absence of the RNA block, T4 polymerase continued polymerization to the end of the DNA template generating a FL 320 nt product (band labeled FL). In the presence of the RNA block, polymerization by T4 DNA polymerase was completely stopped at 220 nt when the polymerase reached the DNA:RNA heteroduplex (band labeled B). In contrast, wild-type R2 synthesized mostly FL product in the presence or absence of the RNA block. Indeed, processivity of the R2 polymerase is actually higher while displacing the RNA block, as there are fewer premature terminations in the duplexed region of the template (minor bands below the FL product). Similar to the wild-type R2 protein, both the -1 and 0 region mutants synthesized similar levels of FL product with and without the RNA block, suggesting there was no reduction in their ability to displace RNA during the polymerization reaction.

### Target primed reverse transcription

**Figure 6.**
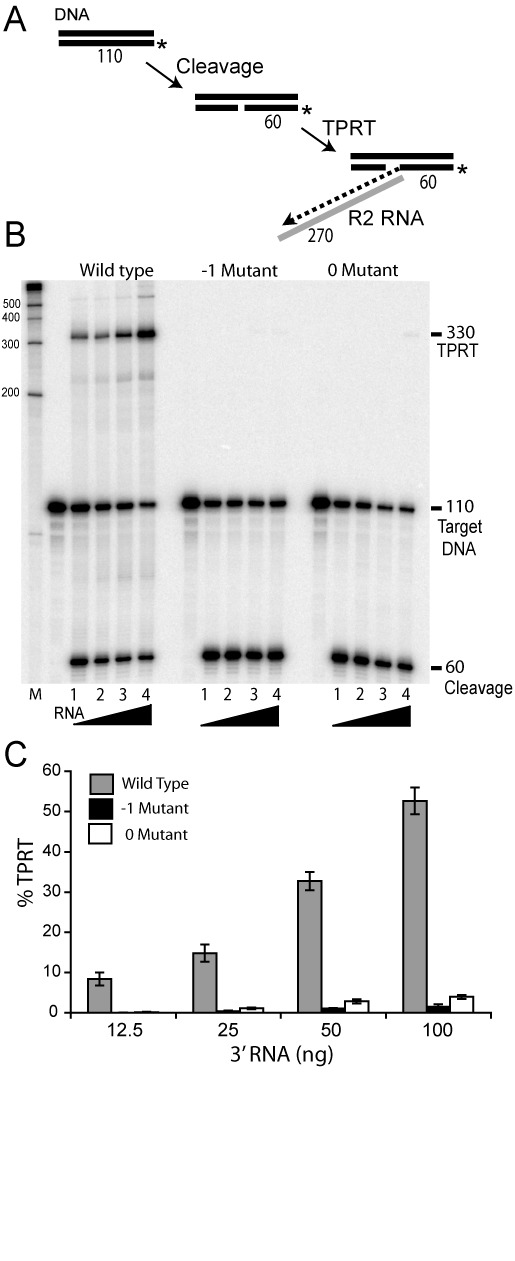
TPRT assay. (A) Diagram of the TPRT reaction. After bottom (first) strand cleavage of the DNA target 5′ labeled on the bottom strand, the R2 protein can use the 3′ end of the 60 nt fragment to prime reverse transcription of RNA corresponding to the 3′ UTR of the R2 element. The combined length of the 60 nt DNA primer and the 270 nt cDNA (dotted line) is 330 nt. (B) Phosphoimager scan of the TPRT reaction for wild-type, -1 and 0 mutant proteins separated on a denaturing 8% polyacrylamide-urea gel. The R2 proteins (4 ng) were first pre-incubated with increasing amounts of 3′ RNA (lane 1, 12.5 ng; lane 2, 25 ng; lane 3, 50 ng; lane 4, 100ng) for 5 min at room temperature. A 110-target DNA (4 ng) 5′ end-labeled on the bottom strand was then added to the reaction and incubated for 20 min at 37°C. (C) Quantitation of the TPRT product generated in three TPRT assays by the wild-type, -1 and 0 mutant proteins.

The critical step in the R2 integration reaction is the utilization of the nick on the bottom strand of the DNA target site to prime reverse transcription (TPRT) (Figure 1). Unlike a simple primer extension reaction, binding of the RNA template in this reaction is highly specific. The only templates that can be used for TPRT are RNAs that contain the 250 nt 3′ UTR of the R2 element. Priming of reverse transcription by the DNA nick does not require complementarity between the RNA template and the DNA target (5,6). To test the ability of the -1 and 0 region mutant proteins to conduct the TPRT reaction, the same 5′ end labeled DNA target site used to test DNA cleavage (Figure 3) was incubated with the R2 protein in the presence of a 270 nt RNA containing the 3′ UTR region of the R2 element and dNTPs to allow reverse transcription. As diagramed in Figure 6A initial cleavage of the bottom strand of the DNA substrate results in a labeled 60 nt fragment, and the utilization of this nick to prime reverse transcription lengthens this DNA to 330 nt if polymerization continues to the end of the input RNA. Because the assay was designed to measure the ability of the protein to bind RNA, the amount of R2 protein in the assay was held constant at a level that resulted in 50% cleavage of the target site and the amount of R2 RNA was increased.

As shown in Figure 6B, wild-type R2 generated significant levels of TPRT product at all RNA concentrations. On the other hand, the -1 and 0 region mutants generated only low levels of TPRT products at the highest RNA levels. Quantifying the amount of label present in the TPRT band relative to the level of cleavage product revealed that the -1 mutant had less than 5% of the wild-type TPRT activity, and 0 mutant had less than 10% of the wild-type TPRT activity (Figure 6C). Because the mutant proteins were readily able to cleave the bottom DNA strand of the target site and were capable of reverse transcription once a template was primed (Figure 5), this assay suggested that the mutant proteins were either unable to bind the RNA, or unable to properly position the 3′ UTR RNA adjacent to the cleaved DNA to allow priming.

### Binding of RNA to the R2 protein

**Figure 7.**
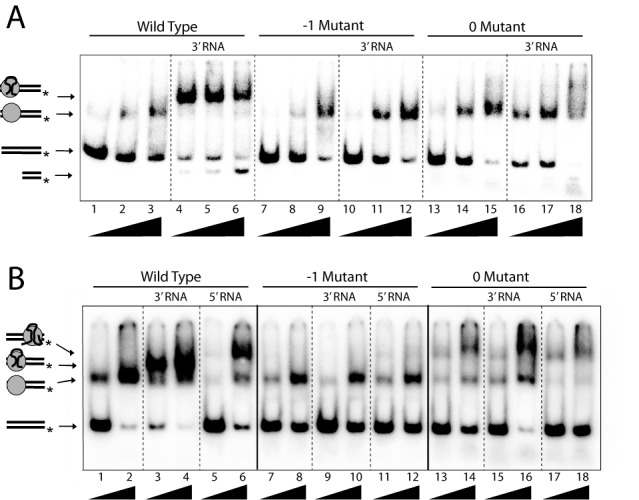
EMSA of the R2 proteins binding to RNA in the presence of the target DNA. (A) The 110-target DNA (4 ng) was 5′ end-labeled on the bottom strand and incubated with increasing amounts of the R2 proteins (lane 1, 1ng; lane 2, 2ng; lane 3, 4 ng) in the absence of 3′ RNA (treated with 1 μg of RNAse A to remove RNA that may co-purify with the R2 protein) or in the presence of 3′ RNA (100 ng). Shown at left are diagrams of the DNA, DNA:protein and DNA:protein:RNA complexes formed in the incubations (4,20). (B) EMSA assays conducted in the presence of 1 μg of RNAse A, 100 ng of 3′ RNA or 100 ng 5′ RNA in the presence of two concentrations of protein (lane 1, 2 ng; lane 2, 4 ng). Assays in B were conducted in the presence of 0.5 mM EDTA to prevent DNA cleavage by the R2 protein.

EMSA were next performed to directly determine whether failure of the TPRT assay was a failure of the mutant R2 proteins to bind the 3′ UTR RNA or a failure to position the RNA properly for TPRT. These gel shift experiments were conduct only with R2 RNAs, as it is difficult to detect wild-type R2 protein binding to non-R2 RNA (4,6,9,20). We have also previously shown that gel shift assays to monitor RNA binding are most sensitive when conducted in the presence of the DNA target (9,20). The affinity of the R2 protein for both the DNA target and R2 RNA substrates greatly increases in the presence of the other nucleic acid. As shown in Figure 7A, in the absence of RNA, incubation of the wild-type protein with the 110 bp DNA target site generated increased levels of shifted complexes as the protein concentration was increased (lanes 1–3). This shifted band corresponds to protein bound to the DNA immediately upstream of the insertion site (diagram at left) (20,28). If, however, the R2 protein was pre-incubated with an excess of the 270 nt 3′ UTR RNA to allow protein-RNA binding, and then the 110 bp DNA target site was added, maximum levels of a super-shifted complex (protein:RNA:DNA) were formed even at the lowest protein concentration (lane 4). This finding confirmed that the presence of 3′ UTR RNA significantly increases the affinity of the R2 protein for the DNA target (20).

Shift assays done with the -1 and 0 mutations revealed target DNA binding in the absence of RNA at levels similar to that observed for the wild-type protein. In the case of the 0 mutant, higher levels of more slowly migrating bands were observed indicating a greater tendency for additional protein subunits to bind to the DNA target at the highest protein concentration (lane 15). In the presence of the R2 RNA, the shifted complexes observed for the -1 mutant were identical to those observed for the protein only assays, suggesting a greatly reduced ability of this protein to bind RNA. In the case of the 0 mutant protein, no RNA-induced super-shifted band was observed at low protein concentrations also consistent with a reduced ability of this protein to bind RNA. However, a range of slower migrating bands was observed at higher concentrations of protein in the presence of RNA compared to the absence of RNA (compare lanes 15 and 18), suggesting a residual level of RNA binding was still possible in the 0 region mutation.

In addition to the ability of the R2 protein to bind RNA corresponding to the 3′ UTR of the R2 element, the R2 protein can also bind a 300 bp region of RNA from near the 5′ end of the R2 element (4). This 5′ RNA binding plays an important role in the integration reaction, as it greatly increases the affinity of the R2 protein for target DNA sequences immediately downstream of the insertion site (4) (Figure 1). Gel shift assays for the binding of the R2 protein in association with either the 3′ or 5′ RNA are shown in Figure 7B. Unlike the assays in Figure 7A, these assays were conducted in the presence of EDTA to prevent complications associated with DNA cleavage. Double-stranded DNA cleavage of the target DNA in the presence of RNA can be readily seen with the wild-type protein on the native gel shown in Figure 7A (lanes 4–6).

As shown in lanes 3–6 of Figure 7B the wild-type protein formed specific super-shifted complexes on the target DNA with both the 3′ and 5′ RNAs. The 5′ RNA is not bound as readily as the 3′ RNA at low concentrations and the specific complex formed at high concentrations of 5′ RNA migrates somewhat slower than the complex with the 3′ RNA. In contrast to the wild-type protein, the -1 mutant showed little evidence of super-shifted bands in the presence of either 3′ or 5′ RNA, suggesting the inability of this protein to bind both RNAs. In the case of the 0 mutant, slower migrating complexes were again detected in the presence and absence of RNA suggesting that multiple protein subunits are binding to the DNA target. These slower moving complexes were most abundant in the presence of 3′ RNA (compare lanes 14, 16 and 18), again consistent with low levels of RNA binding to the 0 mutant.

### Second strand DNA cleavage

**Figure 8.**
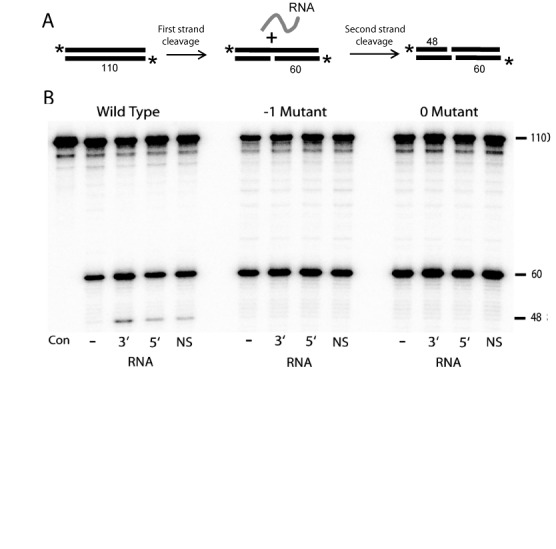
Second-strand DNA cleavage assay. (A) Diagram of the assay. Cleavage of the bottom DNA strand occurs first and does not require RNA. Cleavage of the top strand occurs with reduced efficiency and only in the presence of RNA (3). The 5′ ends of both DNA strands are labeled to independently monitor both cleavage reactions. (B) Phosphoimager scan of the cleavage reactions for wild type, -1 and 0 mutant proteins separated on a denaturing 8% polyacrylamide-urea gel. The 5′ end labeled 110-target DNA (4 ng) was incubated with each protein (4 ng) in the presence of either 1 μg of RNase A (-), 3′ RNA (3′) (100 ng), 5′ RNA (5′) (100 ng) or unrelated vector RNA (V) (100 ng) for 15 min at 37°C. (Con) control cleavage reaction performed in the absence of R2 protein.

Cleavage of the two strands of DNA at the R2 insertion site is conducted by different R2 subunits (Figure 1) (4). Protein bound immediately upstream of the insertion site cleaves the bottom DNA strand, while protein bound downstream of the insertion site cleaves the top DNA strand. Cleavage of the top strand is much less efficient and only occurs if RNA is present in the reaction (3). The presence of the RNA does not appear to play a direct catalytic role in the DNA cleavage reaction as any RNA sequence will act as a stimulus (3). Any RNA sequence can promote binding of the R2 protein downstream of the insertion site which enables top strand cleavage as long as R2 protein is also bound upstream of the insertion site. The 5′ R2 RNA is unique, however, in that it can promote downstream binding of the protein in the absence of upstream binding (4). To assay whether the -1 and 0 mutant proteins were able to bind RNA well enough to enable top strand cleavage, the 110 bp DNA target was 5′ end labeled on both the top and bottom strands (Figure 8A). Cleavage of the bottom strand results in a labeled 60 nt fragment while cleavage of the top strand results in a labeled 48 nt fragment. Cleavage was assayed in the presence of 3′ RNA, 5′ RNA or non-specific RNA (plasmid vector sequences). As shown in Figure 8B, wild-type protein was able to cleave the top strand in the presence of all three types of RNA. With a level of detection of ∼5% that of wild-type protein cleavage, both the -1 and 0 mutants were unable to cleave the top strand in the presence of any of the RNAs.

## DISCUSSION

Comparison of R2 protein sequences from diverse metazoans revealed two conserved regions (0 and -1) upstream of the series of common motifs that can be identified in all reverse transcriptases (21). Analysis of clustered mutations which replace some of the most conserved amino acids in these two motifs suggest these motifs form part of a RNA binding domain of the R2 protein. Mutations in either motif retained the ability of the R2-RT to extend primers annealed to RNA or DNA templates but dramatically eliminated both TPRT activity and the ability of the R2 protein to bind RNA in gel shift assays.

We previously suggested that the 0 motif might enable the R2 protein to bind more extensively to template sequences upstream of the active site of the RT domain, thus, allowing the R2-RT to undergo template jumps and displace an RNA strand annealed to a DNA template (8–10). Evidence for this model can be found in this report, because the 0 mutant had dramatically reduced ability to undergo template jumps with either RNA or DNA templates (Figure 3). However, inconsistent with the previous model the 0 mutant was fully capable of displacing an RNA strand annealed to a DNA template (Figure 5). Because a small reduction in activity of the 0 region mutation was detected in simple primer extension assays, the alterations in this motif may have induced subtle changes in the catalytic domain of R2-RT. On the other hand, the -1 motif mutation effectively eliminated all activity of the R2 protein that was known to be dependent upon RNA binding without any detectable effect on the catalytic activity of the RT domain. Thus, we suggest this motif has a high probability of being exclusively involved RNA binding. Much work remains to determine how one region of the R2 protein is able to bind both the 3′ and 5′ regions of the R2 transcript, yet 3′ RNA is positioned to be used as template for TPRT, while the 5′ RNA changes the specificity of the R2 protein for binding to DNA.

The 0 motif can be found in all lineages of non-LTR retrotransposons (25,29). For example, in mammalian L1 elements, this motif has been termed the Z segment (29,30). L1 encoded proteins in all mammals contain minor variants of the sequence EL-9aa-SPGPDGF, highly similar to the sequences conserved in all R2 elements (Figure 2). While the studies reported here suggested that mutations in this motif result in only a slight reduction in the ability of the R2-RT to extend a primer, mutagenesis of the human L1 protein suggested this motif was required for reverse transcription on poly (A) and poly(G) templates (29). However, there are two concerns regarding this previous study of the L1 protein. First, the L1 protein has proven to be extremely difficult to isolate, thus the *in vitro* assays were conducted with crude cell lysates. Because no other activity could be assayed, there was no means to insure that the mutant L1 proteins were stabile. Second, the L1-RT activity being assayed was not dependent upon the addition of a primer, thus, there is uncertainty as to how DNA synthesis was initiated in these assays. Based on the experiments presented here and the similarity to telomerase and the RT of mobile group II introns described below, we suggest that RNA binding is an important role of the 0 motif in most other non-LTR retrotransposons including L1.

The RNA binding motifs N-terminal to the universal RT motifs have been identified in both mobile group II introns and in telomerases. Group II introns have a conserved motif (labeled RT0 in ref. 31) with sequence similarity to that of the 0 motif described here. Central to this similarity most group II introns contain a G-hydrophobic-D-G motif similar to the conserved residues in the R2 protein that were mutated in this report (Figure 2). However, using a high-throughput screen for mutations of the L1.LtrB intron encoded protein that detected reduced binding to intron RNA, the protein regions most resistant to change were located to either side of the conserved 0 motif (19). The region N-terminal to the motif 0 of L1.LtrB was suggested to be the major component of the RNA binding domain. This N-terminal region, which could be functionally similar to motif -1 of the R2 protein, is only 35 amino acids in length in L1.LtrB. In the case of R2, the N-terminal domain appears larger as the amino acid mutations that eliminated motif -1 functions in R2 are over 70 residues upstream of motif 0 (Figure 2).

The more extensive characterization of RNA binding by telomerase has included X-ray diffraction studies of the isolated RNA binding domain as well as the complete telomerase (18,32). Two regions of the protein appear to be involved in binding to the telomerase RNA. The T motif which like R2 and group II introns is located immediately upstream of the common RT motif and a second domain, CP, which is located about 135 amino acids upstream of T. The T and CP motifs interact with regions of the telomerase thumb effectively encircling the RNA template (18). The T motif contains several conserved, large hydrophobic residues and forms a narrow deep cleft in the protein. The T motif has no obvious sequence similarity to the 0 motif seen in R2 or L1-LtrB. The CP motif forms a shallow cleft that is less conserved in sequence, and similar to the -1 motif of R2 has an accumulation of interspersed hydrophobic and charged residues.

Phylogenetic comparisons based on the sequences of the conserved domains in all reverse transcriptase-like enzymes, suggest that non-LTR retrotransposons, telomerase and group II introns are phylogenetically related and separate from the reverse transcriptases of LTR retrotransposons and retroviruses (16,21,25,33). The ability of each of these enzymes to specifically bind an RNA and then to use that RNA as a template for reverse transcription primed by the 3′ end of DNA provides additional evidence for the close evolutionary relationship of these enzymes. Future studies of non-LTR retrotransposon RTs should use telomerase and group II introns as guides for structure/function studies.
